# Dr. O'Leary, on Yellow Fever

**Published:** 1806-12

**Authors:** Edmond O'Leary

**Affiliations:** Assistant Surgeon, 52d Regiment. London


### *To the Editors of the Medical and Physical Journal.*

Gentlemen,

I Feel great pleasure in being able to communicate to you the remarkable success which has attended the use of the affusion of cold water in the yellow fever, a remedy which has rescued many valuables lives from a disease which has spread such destruction through our army in the West Indies.

A few days after my arrival at Barbadoes, from Europe, in November last, I was ordered by the Inspector of the Medical Department, to repair to Antigua, and attend the sick of the 70th regiment, which I found amounted to about one hundred. They were chiefly affected with the yellow fever, and the mortality was exceeding great.

I was extremely anxious to give a fair trial to the cold affusion in a few cases, notwithstanding its having been unsuccessfully unsuccessfully employed by an assistant surgeon of the 70th regiment, a short time previous to my arrival there, which can only be attributed to his having used it indiscriminately in every stage of the disease, consequently it must often have accelerated the patient's dissolution; I was led to its use from the similarity of the symptoms of the yellow fever, in its incipient state, to some cases which I have seen most successfully treated by Dr. Home, in the Edinburgh Infirmary.

The soldiers of the 70th regiment were at first generally seized with slight shiverings, soon followed by intense thirst; heat of body much augmented; pulse from 120 to 140 in the minute, and full; face flushed; nausea and vomiting of a green bilious fluid; a violent pain of the forehead and balls of the eyes, sometimes with a dilatation of the pupils. On the fourth or fifth day, the skin exhibited a yellow colour, but it was by no means an universal occurrence. A yellowness of the eyes, to a remarkable degree, appeared in most cases.

As soon as the patient was carried to the Hospital, I directed two buckets full of cold water from the cistern, which is under ground, to be poured over him; he was then laid in bed, and experienced the greatest possible relief. The heat of skin was considerably diminished, frequency of pulse much reduced, pain of forehead and eye balls constantly abated, and, mostly, a gentle perspiration succeeded. In a short time, however, all the symptoms returned, with the heat of skin, but in a milder form; the affusion was again applied, and occasioned the most grateful sensation. Several orderly men sat up during the night, in the hospital, with directions to repeat the affusion when the heat became greater than natural, but unattended with perspiration.

In the course of two days the morbid heat, and excessive frequency of pulse, with which all the other symptoms were connected, were so completely subdued by repeated affusions, that I seldom found it necessary to direct their use more than two or three times on each of the succeeding days of the fever; frequently, at that period, ablution of the breast, hands, and feet, answered the purpose equally well. By this mode of treatment only one case of thirty-four, in which it was first employed, terminated fatally. So sensible were the men of its superior efficacy to any other remedy, and of the relief obtained from it, that in my absence they frequently intreated the officers, whose duty duty led them to visit the hospital, to have it repeated on them.

One morning, on my return from visiting the patients in the hospital, with Mr. Ireland, the assistant surgeon, we were both violently seized with the usual symptoms of the yellow fever. Mr. Ireland was first attacked, and immediately used the warm bath; he was reduced to so low a state of debility by the fever, that a medical board recommended him for leave to return to Europe, as the only means of recovering his health. I considered the cold affusion as a remedy, from which alone there appeared any probability of my surviving the disorder; I determined therefore to employ it to the fullest extent, having seen its use attended with the most extraordinary success in the hospital of the 70th regiment.

As soon as the hot stage was fully formed, I was placed in a tub, and had two buckets of water from the cistern poured over me, by Capt. Elliott, of the 70th regiment, whose attention to me, during my illness, can never be erased from my memory. I was then dried with towels, and laid in bed. It is impossible to express the pleasing sensations which follow the affusion in this fever. I was most completely relieved from the burning heat, which a few minutes before most severely oppressed me, and the pain of the head was much less severe; the relief, however, was but temporary; I was soon after distressed with a recurrence of excessive heat, and the affusion was again resorted to, which was followed by some refreshing sleep.

The fever continued, with severity, for seven days, and the affusion was had recourse to thirty-seven times during that period, by Captain Elliott; I was also repeatedly washed by my own servant with lime juice, or vinegar and water. The thirst was most excessive; no drink was so grateful to me as cold water, of which I must have used a vast quantity, for no sooner was the vessel removed from my lips than I entreated to have it again, as my lips and tongue felt nearly as parched as before I had drank.

During my illness a surgeon attended the hospital, who was an utter enemy to the cold affusion in the yellow fever. The instant a soldier was taken ill with it, the warm bath was ordered for him, and almost every case terminated fatally; the regiment frequently lost five men a day, and many, when attacked with the fever, were very unwilling to go to the hospital, and were treated with the affusion by the officers in the barrack rooms.

As

As soon as I was sufficiently recovered to do duty again, I took charge of the hospital, with Mr. Rozea, of the staff, who had a high opinion of this practice. The yellow fever was the principal disease we had to combat; we resumed the use of the affusion, in such cases as we supposed advantage might be derived from it; some cases occurred where hepatitis was combined with the fever, in such cases it was omitted, as we were confident the affusion would aggravate the former; very few recovered who were affected in this manner.

In three cases the fever, when fully formed, has been cut short by the affusion on the first day; these I shall more particularly relate.

Cornelius Coonahan, admitted into the hospital, Dec. 3, 1806, complained of considerable pain of the head and back; countenance greatly flushed; pulse 140 in the minute, and full; skin dry, and extremely hot; pupils of the eyes dilated; tongue red; thirst and nausea excessive; previous to admission had slight shiverings. Two buckets of water were poured over him; in a few minutes after, pulse was 120; heat, thirst and nausea much diminished, and a gentle sleep succeeded. In the evening the fever was again severe; pulse at 130; the affusion was repeated with the happiest effects. About 9 P. M. he had a slight return of thirst, and heat of skin; the affusion was once more had recourse to; pulse fell to 80 in a minute; heat to nearly the standard of health; pain of head quite gone; and on the following day was free from complaints.

Michael Davy, admitted Dec. 5, 1805; pulse was 125, and very full; thirst urgent; heat of skin very great; tongue dry and parched; pain of head and eye balls severe; had two buckets of water thrown over him; pulse fell to 108; heat sunk considerably. In a short time heat and thirst were again severe, and he most eagerly demanded a repetition of the affusion, which was given him, and soon after the disease was completely removed.

James Kelly, admitted Dec. 10, 1805, with increased heat of skin; pulse 116; pain in the eye balls; vomiting of a green fluid; tongue red, and much thirst. He was stripped perfectly naked, placed in a tub, and three buckets of water were poured over him. The usual pleasing effects were here particularly remarkable; pulse was reduced in frequency, heat to the natural standard, a gentle diaphoresis succeeded, and the disease no more returned.

I pointed out these cases particularly to Lieutenant-Colonel lonel Ross, who was so convinced of the great utility of that inestimable remedy, the cold affusion in the yellow fever, that he immediately erected a shower bath at the hospital.

The officers, when taken ill, at their own particular desire, were treated with the affusion; on one it was repeated twenty-one times in twenty-four hours. During the time the yellow fever prevailed in the 70th regiment, the affusion has been used in one hundred and eighty-five cases, of which number five only terminated fatally. Four of them concealed their complaints, and were not carried to the hospital until the third day of the disease.

I have constantly paid particular attention in only employing it in such stages of the fever as have been recommended by Dr. Currie, where the heat of the skin was greater than natural, and no sense of chilliness, or much perspiration, was present; sometimes the saline mixture was administered a few minutes after it; but, in general, a complete recovery was effected by the sole addition of some purgatives, which were always necessary during the fever; at first with a view of completely evacuating the intestinal canal, and afterwards of carrying off the bile which is most abundantly secreted; a solution of some cathartic neutral salt, with the addition of carbonate of magnesia, answered this purpose most effectually. The sal. muriate of mercury has been extensively employed in the West Indies with this intention, but I have never found it answer; in every instance, owing to the excessive irritability of the stomach, it has excited vomiting.

The patients who died, on whom the cold affusion was employed, were affected from the very commencement with a violent degree of vomiting; oue indeed, during a considerable time prior to the attack of fever, was daily in the habit of throwing up a vast quantity of frothy bile; effervescing draughts, with the addition of æther, or a few drops of the tincture of opium, were instantly rejected; blisters to the stomach afforded little relief. A short time before death they were seized with delirium, and from that instant the vomiting ceased; the appearances on dissection seemed to account for this.

The stomach contained a black viscid fluid, which I supposed to be putrid bile; about the cardia a high degree of inflammation was observable, the villous coat was abraded in many parts; the small intestines, particularly the duodenum, exhibited a number of red lines, and the villous coat coat was separated from the others; the liver appeared much distended, and the gall bladder contained a quantity of bile of a yellow colour, and extremely thick.

From the appearances after death, I concluded the delirium arose in consequence of a rupture of the internal coat of the stomach, from the well known sympathy existing between that organ and the brain; and when that occurrence took place, the inverted action of the stomach must have necessarily ceased: these symptoms appeared in each of the five cases which terminated fatally.

I cannot imagine this fever was contagious, but rather suppose it was produced by marsh exhalations; for during the time the 70th regiment, stationed at St. John's, in the vicinity of a marsh, were affected with it, the 68th regiment, at Monk's Hill, a distance only of eight miles, and the 96th regiment and artillery at the Ridge, were healthy. I am, &c.

EDMOND O'LEARY, M. D.
Assistant Surgeon, 52d Regiment.

*London, Oct.* 10, 1806.

